# Police work stress and occupational burnout: the chain mediating roles of work interference with family and affective work rumination

**DOI:** 10.3389/fpsyg.2026.1955185

**Published:** 2026-09-08

**Authors:** Jianxing Fu, Chunhua Chen, Xianghui Lai

**Affiliations:** Fujian Police College, Fuzhou, China

**Keywords:** affective work rumination, occupational burnout, police work stress, serial mediation, work interference with family

## Abstract

**Background:**

Work-related strain may extend beyond the workplace by disrupting family life and sustaining emotionally distressing thoughts about work during off-job time. This study tested whether the association between police work stress and occupational burnout included a serial indirect component through work interference with family (WIF) and affective work rumination (AWR).

**Methods:**

A cross-sectional survey was completed by 1,464 police officers serving across nine cities in southeastern China. Structural equation modeling (SEM) was used to estimate the direct association and three prespecified indirect effects involving WIF and AWR.

**Results:**

Bivariate analyses revealed significant positive associations among police work stress, work interference with family (WIF), affective work rumination (AWR), and occupational burnout. Police work stress was significantly and positively associated with overall occupational burnout and its three specific dimensions. In the overall occupational burnout model, the indirect effects through WIF, through AWR, and serially through both were all statistically significant. Dimension-specific analyses showed that the total indirect effect was significant in the personal burnout and work-related burnout models, but not in the client-related burnout model.

**Conclusion:**

The direct association between police work stress and occupational burnout remained significant after WIF and AWR were included in the model. Significant indirect effects were observed separately through WIF and AWR. The serial indirect association through WIF followed by AWR was also statistically significant. Burnout-prevention programs could address organizational stressors alongside family-supportive practices, work–home boundary management, and techniques for reducing affective rumination outside working hours.

## 1 Introduction

Police officers constitute a key occupational group responsible for maintaining social order and public safety. Their occupational mental health is closely related not only to their own physical and psychological well-being but also to the conduct of law enforcement, police-public interactions, and the capacity of police organizations to provide public safety services on a sustained basis (Alves et al., 2023; Queirós et al., 2020). Police officers work in highly structured institutional settings marked by sustained pressure and intensive demands. They may encounter threats of violence, traumatic events, and emotionally demanding interpersonal interactions, while also experiencing heavy workloads, long working hours, bureaucratic procedures, and inadequate organizational support over extended periods (Drew and Williamson, 2025; McCreary and Thompson, 2006; Purba and Demou, 2019). Occupational burnout represents an important manifestation of the cumulative psychological toll associated with sustained exposure to intense occupational stress among police officers (Kristensen et al., 2005). The importance of research on police occupational burnout extends beyond individual fatigue and health impairment. Research has also identified a significant overall positive association between occupational burnout among police officers and symptoms of post-traumatic stress disorder (PTSD) (Ugwu and Idemudia, 2025).

Although previous research has examined occupational burnout extensively and documented several associations among work stress, work interference with family, affective work rumination, and occupational burnout, two more specific theoretical questions remain insufficiently addressed: how work interference with family (WIF) and affective work rumination (AWR) are linked within the association between work stress and occupational burnout once that stress extends beyond the work domain, and whether this sequential pattern is similarly evident across burnout dimensions defined by different referents. Accordingly, the present study examines the direct and indirect pathways linking police work stress to occupational burnout, with particular emphasis on the separate and serial indirect associations involving WIF and AWR. In doing so, it seeks to refine current understanding of burnout-related health impairment and provide an empirical basis for improving occupational-stress interventions, family-support services, and psychological recovery programs for police officers.

### 1.1 The association between police work stress and occupational burnout

Police work stress encompasses officers’ cumulative exposure to a range of occupational stressor events and the strain associated with such exposure. Police stressors are commonly grouped into operational and organizational categories (McCreary and Thompson, 2006). Operational stressors arise directly from policing duties and include exposure to violence or the risk of physical injury, encounters with death and traumatic events, involvement in critical incidents and emergency response, and emotionally demanding interactions with victims, suspects, and members of the public in crisis (Queirós et al., 2020; Violanti et al., 2017). In these situations, officers must remain continuously vigilant, make potentially consequential judgments under time pressure and with limited information, and regulate and display emotions in accordance with professional norms. When recurrent, these cognitive, emotional, and physical demands may be accompanied by stress activation that persists beyond the end of a duty, greater fatigue and emotional exhaustion, and poorer off-job recovery.

Organizational stressors stem from work arrangements and organizational functioning and include heavy workloads, staffing shortages, shift work and long working hours, bureaucratic procedures, role conflict, leadership practices, and inadequate organizational support (Drew and Williamson, 2025; Purba and Demou, 2019). Such stressors are typically embedded in routine policing, occur frequently, persist over time, and remain largely beyond the control of individual officers. They encompass elevated job demands and may coincide with lower job autonomy, less predictable schedules, weaker perceptions of procedural fairness, and fewer support resources, all of which are relevant to officers’ capacity to manage high-risk operational duties and recover afterward. Operational and organizational stressors often co-occur in everyday policing. Their accumulation is characterized by sustained demands on attentional control, emotion regulation, and physical resources, together with restricted opportunities for sleep, family involvement, and off-job recovery.

Occupational burnout refers to a persistent state of exhaustion associated with chronic work stress that has not been adequately alleviated. According to the referent of exhaustion, it can be differentiated into personal burnout, work-related burnout, and client-related burnout (Kristensen et al., 2005). Sustained physical, cognitive, and emotional demands require continued effort. When resources such as adequate staffing, leadership support, and opportunities for recovery are insufficient, this combination is associated with progressive energy depletion and higher levels of occupational burnout (Bakker and Demerouti, 2007; Demerouti et al., 2001). Occupational burnout among police officers should therefore be examined in the context of long-term exposure to occupational psychosocial stressors and the organizational conditions in which such exposure occurs (Sun et al., 2026).

Existing studies have identified both operational and organizational stressors as important risk factors for occupational burnout among police officers (Alves et al., 2023; Purba and Demou, 2019). The highly stressful conditions of police work may therefore be directly associated with occupational burnout (Bakker and Demerouti, 2007; Gutschmidt and Vera, 2022; Lambert et al., 2022). Research involving Chinese police officers has similarly identified work stress as an important risk factor for occupational burnout (Zhou et al., 2024). Taken together, these findings provide a clear theoretical rationale and substantial empirical support for the association between police work stress and occupational burnout (Gomes et al., 2022; Maran et al., 2022).

Importantly, Blomqvist Mickelsson et al. (2026) reported no significant overall association between burnout and use of force when the burnout dimensions were combined. Dimension-specific analyses, however, showed a small positive association between depersonalization and use of force, whereas neither emotional exhaustion nor personal accomplishment was significantly associated with use of force. These findings suggest that reliance on a single total burnout score may obscure dimension-specific associations with external outcomes and underscore the need to examine individual burnout dimensions separately. Accordingly, we propose the following hypothesis:

*H1*. Police work stress is positively associated with occupational burnout.

### 1.2 Police work stress, work interference with family (WIF), and occupational burnout

Work interference with family (WIF) refers to the extent to which the time demands, strain, and behavioral expectations associated with an individual’s work role hinder the fulfillment of family responsibilities, participation in family activities, and maintenance of family relationships (Greenhaus and Beutell, 1985; Netemeyer et al., 1996). As the work-to-family direction of interrole conflict, it is closely related to occupational stress. Workload, long working hours, role stress, and role conflict have consistently been identified as important work-related antecedents of work–family conflict (Byron, 2005; Michel et al., 2011). Police work is characterized by unpredictable demands, shift schedules, exposure to danger, and substantial public accountability, all of which place sustained demands on officers’ time, emotional energy, and physical resources. Organizational directives, overtime duties, interactions with the public, and case-related pressures may consequently spill over into family life, limiting officers’ ability to meet caregiving responsibilities, communicate effectively with family members, and participate in shared activities (Hall et al., 2010; Sadiq, 2022). Conservation of resources (COR) theory proposes that individuals seek to acquire, retain, and protect valued resources, whereas prolonged stress threatens or depletes those resources and increases competition between life roles (Grandey and Cropanzano, 1999; Hobfoll, 1989). Viewed from this perspective, officers experiencing higher levels of work stress may be more likely to perceive that their occupational responsibilities intrude upon family life. Time, energy, and emotional resources devoted to work become less available for family responsibilities and relationships, thereby increasing WIF.

WIF may subsequently be associated with occupational burnout. Research on the consequences of work–family conflict has consistently linked the work-to-family direction of conflict to psychological strain, emotional exhaustion, job dissatisfaction, and burnout (Allen et al., 2000; Amstad et al., 2011; Kossek and Ozeki, 1998). Evidence from police samples further indicates that work–family conflict may help account for the relationship between job demands and emotional exhaustion (Griffin and Sun, 2018; Hall et al., 2010; Lambert et al., 2019). When work-related time pressures, strain, and emotional demands persist after a shift has ended, family life may offer fewer opportunities for recovery, social support, and emotional replenishment (Thompson et al., 2005). Reduced recovery in the home domain may, in turn, allow fatigue and psychological strain to accumulate over time (Bennett et al., 2018; Moreno-Jiménez et al., 2009; Sonnentag et al., 2010). Complementing the resource-loss perspective, the JD–R model proposes that prolonged exposure to demanding working conditions contributes to burnout through an energetic depletion process (Bakker and Demerouti, 2007; Demerouti et al., 2001; Halbesleben, 2006). When work intrudes upon family life, restricted opportunities for recovery and reduced access to family support may intensify this process (Halbesleben, 2006). WIF may therefore constitute an important cross-domain pathway through which police work stress is associated with occupational burnout.

Accordingly, we propose the following hypothesis:

*H2*. Work interference with family (WIF) mediates the association between police work stress and occupational burnout.

### 1.3 Police work stress, affective work rumination (AWR), and occupational burnout

Affective work rumination (AWR) refers to recurrent thoughts about work-related events during nonwork time that are accompanied by negative emotional experiences, such as tension, distress, irritability, fatigue, and worry (Cropley et al., 2012; Querstret and Cropley, 2012). Rather than ending when the workday is over, AWR allows occupational strain to persist beyond working hours by prolonging stress-related activation and impeding recovery from job demands (Brosschot et al., 2006; Sonnentag and Fritz, 2015). As a maladaptive form of work-related cognition, it is often accompanied by negative emotional arousal, attentional fixation, and continued depletion of psychological resources (Cropley et al., 2012; Jimenez et al., 2022). Police work imposes substantial cognitive and emotional demands arising from operational incidents, case management, public complaints, organizational evaluations, shift arrangements, and accountability pressures (Maran et al., 2022; McCreary and Thompson, 2006; Purba and Demou, 2019). When tasks remain unfinished, involve potential threats, or carry serious consequences, work-related concerns may continue to occupy officers’ attention during nonwork time. Officers may repeatedly revisit operational details, their personal responsibilities, and the possible consequences of work events (Syrek et al., 2017). Previous research has consistently associated higher job demands with greater work-related rumination. Prolonged exposure to demanding working conditions may also make it more difficult for employees to detach psychologically from work, thereby increasing their susceptibility to affective rumination (Kinnunen et al., 2017; Perko et al., 2017). Research involving Chinese police officers has similarly found a positive relationship between work-related rumination and emotional exhaustion, highlighting the resource-depleting implications of repetitive work-related thinking in high-pressure policing contexts (Pei and Wu, 2022). Officers experiencing higher levels of police work stress may therefore be particularly likely to engage in AWR after their shifts.

AWR may, in turn, be associated with occupational burnout. By extending work-related cognitive and emotional activation into periods otherwise available for rest, persistent rumination may increase fatigue, impair sleep quality, and contribute to the accumulation of negative affect (Querstret and Cropley, 2012; Sonnentag et al., 2010). Incomplete recovery may leave individuals with fewer psychological resources to manage subsequent work demands, increasing their vulnerability to personal, work-related, and client-related burnout (Kristensen et al., 2005; Maslach et al., 2001). Meta-analytic evidence further indicates that negative work-related thoughts are more strongly related to burnout, health problems, and impaired occupational functioning than constructive forms of work reflection, with affective rumination representing a prominent form of maladaptive work-related cognition (Jimenez et al., 2022). Evidence from police samples also suggests that rumination is implicated in the relationship between occupational burnout and posttraumatic stress symptoms. This finding further illustrates how repetitive processing of stressful occupational experiences may intensify psychological strain in policing contexts (Ogińska-Bulik and Juczyński, 2021).

AWR may therefore constitute a cognitive–affective pathway through which police work stress is associated with occupational burnout.

Accordingly, we propose the following hypothesis:

*H3*. Affective work rumination (AWR) mediates the association between police work stress and occupational burnout.

### 1.4 The serial mediating roles of work interference with family (WIF) and affective work rumination (AWR)

The Effort–Recovery model provides an important framework for explaining how occupational stress carries over into nonwork time. It proposes that meeting job demands requires sustained investment of psychological and physiological resources and that post-work recovery can occur only when the functional systems mobilized during work are no longer exposed to continued demands (Meijman and Mulder, 1998; Sonnentag and Fritz, 2007). COR further maintains that individuals strive to preserve and replenish finite resources, whereas simultaneous demands from work and family compete for the same resource pool and may consequently generate resource loss and interrole strain (Ten Brummelhuis and Bakker, 2012; Hobfoll, 1989). From this perspective, WIF captures the role-based depletion that occurs when occupational stress spills over into family life, whereas AWR represents the cognitive–emotional process through which work-related activation persists during nonwork hours (Brosschot et al., 2006; Cropley et al., 2012; Querstret and Cropley, 2012). These two mechanisms may operate sequentially rather than independently. When work encroaches on family life, officers may continue to face delayed family activities, disrupted responsibilities, and unresolved emotional tension during time otherwise available for recovery. Such interference can undermine psychological detachment and diminish the restorative value of the family domain (Edwards and Rothbard, 2000; Geurts and Demerouti, 2002; Sonnentag and Fritz, 2015). Under these conditions, work-related cues may become increasingly intertwined with family conflict, perceived failure to meet family obligations, and negative affect. Officers may therefore remain mentally preoccupied with work events after leaving the workplace, repeatedly revisiting them with feelings of tension, irritation, and distress.

Previous research has consistently associated work–family conflict with psychological strain, job dissatisfaction, emotional exhaustion, and occupational burnout (Allen et al., 2000; Amstad et al., 2011). Because work interference with family arises when job demands, working hours, and occupational strain impede family responsibilities, it may erode the family domain’s capacity to support recovery while sustaining attention to work-related stressors (Greenhaus and Beutell, 1985; Netemeyer et al., 1996). AWR may then prolong stress-related psychological and physiological activation, preventing adequate resource replenishment during nonwork time and allowing fatigue, negative affect, and burnout to accumulate (Brosschot et al., 2006; Querstret and Cropley, 2012; Zijlstra and Sonnentag, 2006). Consistent with the JD–R model, chronic job demands contribute to burnout through resource depletion, and this process may be intensified when family-based recovery is disrupted and AWR remains elevated (Bakker and Demerouti, 2007; Demerouti et al., 2001). This sequence may be particularly salient in policing. Intensive operational duties, shift work, emergency response requirements, and organizational accountability may initially restrict the time and resources available for family life. The resulting lack of recovery within the home domain may then foster persistent negative thinking about work (Griffin and Sun, 2018; Hall et al., 2010; Querstret and Cropley, 2012).

The proposed ordering of WIF before AWR is consistent with evidence suggesting that repetitive thought may be implicated in the association between work–family conflict and poorer health outcomes (Davis et al., 2017). Police work stress is associated with difficulties fulfilling family responsibilities and reduced participation in family activities. The accompanying role strain, sense of unfinished obligations, and negative experiences may keep work-related cues salient during family time; this continuing salience may, in turn, be associated with greater AWR (Griffin and Sun, 2018; Hall et al., 2010; Querstret and Cropley, 2012). The sustained cognitive and emotional engagement represented by AWR is further associated with higher levels of occupational burnout. WIF and AWR may therefore jointly constitute a role-based and cognitive–affective sequence through which police work stress is linked to occupational burnout.

Accordingly, we propose the following hypothesis:

*H4*. Work interference with family (WIF) and affective work rumination (AWR) serially mediate the association between police work stress and occupational burnout.

**Figure 1:**
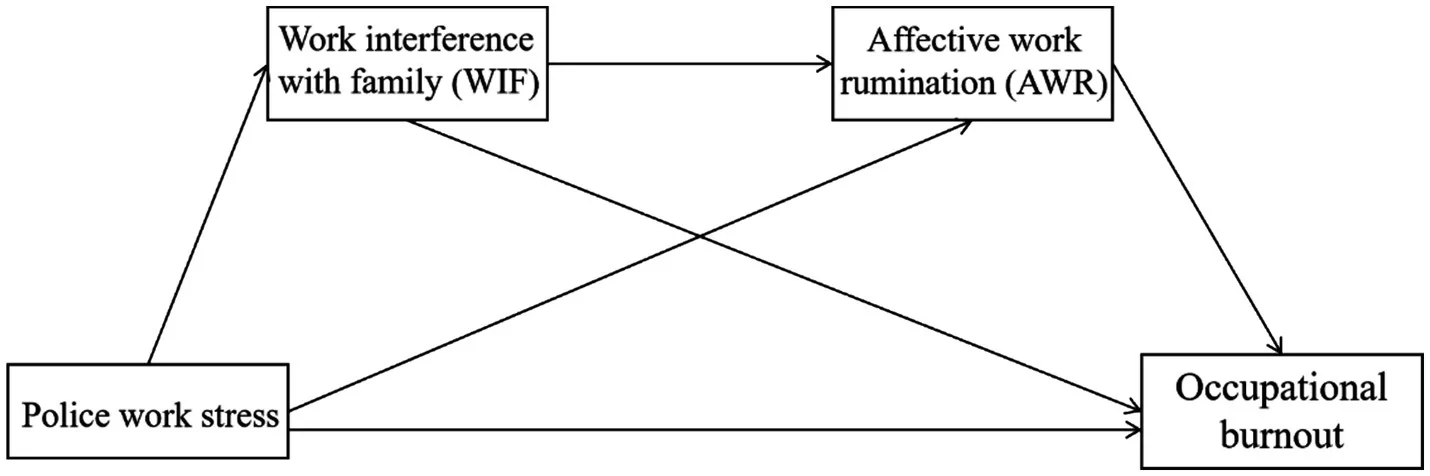
The hypothesis model.

To test H1–H4, the present study developed the overall hypothesized model shown in Figure 1. The model examines the association between police work stress and occupational burnout and the intermediary roles of WIF and AWR.

## 2 Methods

### 2.1 Participants

The study population comprised active-duty officers serving in nine cities within a province in southeastern China. Each municipal police organization designated a local coordinator with psychology-related training or experience. Their role was limited to survey distribution, and they were unable to identify which invitees ultimately participated. Participation was entirely voluntary, and invitees who declined or discontinued participation were not replaced.

Before the survey was administered, all local coordinators received standardized instructions from the research team in collaboration with the participating police organizations. The instructions addressed recruitment, survey administration, informed consent, and the protection of participant information. Prospective participants were required to review an electronic information and consent page before accessing the questionnaire. This page explained the purpose and procedures of the study, the voluntary nature of participation, the right to discontinue participation at any time, the measures used to protect confidentiality, and the available channels for obtaining assistance or submitting concerns or complaints. No directly identifying information was requested in the questionnaire, and neither the participating police organizations nor any external party could link individual responses to particular officers.

Data were obtained through anonymous self-report questionnaires. Ethical approval was granted by the Research Ethics Committee of Fujian Police College (approval no. FPCER-202601-004). The study procedures complied with the principles of the Declaration of Helsinki and its subsequent amendments.

Of the 1,682 officers who submitted questionnaires, 218 cases were removed during data-quality screening because of missing responses, indications of inattentive responding, or uniform selection of the same response option across all items. The final analytic sample therefore consisted of 1,464 officers, accounting for 87.04% of the submitted questionnaires. The participants were, on average, 40.94 years old (SD = 9.67) and had served as police officers for 17.91 years (SD = 10.11). Further demographic information is reported in Table 1.

**Table 1:** Demographics of participants (*N* = 1,464).

Variable	Category	Frequency	Percentage (%)
Gender	Male	1,181	80.67
	Female	283	19.33
Marital history	Never married	267	18.24
	Ever married (including currently married, remarried, divorced, and widowed)	1,197	81.76
Educational level	High school or below	8	0.55
	Junior college	212	14.48
	Bachelor’s degree	1,211	82.72
	Master’s or doctoral degree	33	2.25

### 2.2 Measurement

#### 2.2.1 Police Stress Scale (PCSS)

Participants completed the 50-item Police Stress Scale (PCSS), an instrument specifically developed for assessing occupational stress among Chinese police officers (Chen and Hu, 2012). Its items are organized into six dimensions: work tasks (PCSS1, 9 items), social life (PCSS2, 12 items), power motivation (PCSS3, 8 items), personal competence (PCSS4, 7 items), negative emotions (PCSS5, 5 items), and organizational management and working conditions (PCSS6, 9 items). Collectively, these domains capture stress arising from heavy workloads and non-policing duties, the effects of police work on officers’ family and social lives, workplace power relations and interpersonal conflict, perceived limitations in professional competence, adverse emotional experiences, and unfavorable organizational practices or working conditions.

The PCSS uses a two-stage response format. For each potential stressor, officers first reported whether they had encountered it by selecting “yes” or “no.” When a stressor was endorsed, they rated its perceived impact on a five-point Likert-type scale anchored by “no impact” and “extremely severe impact.” Higher aggregate scores represent greater police work stress.

Previous research has supported the reliability and factor structure of the PCSS (Wu, 2022; Zheng, 2025). In the present study, confirmatory factor analysis (CFA) of the original 50 items supported the hypothesized correlated six-factor structure: χ^2^ = 3,943.332, df = 1,160, χ^2^/df = 3.399, CFI = 0.949, TLI = 0.946, RMSEA = 0.040, and SRMR = 0.036. The total scale showed excellent internal consistency (Cronbach’s α = 0.971; McDonald’s ω = 0.971). Reliability was also high across the six dimensions, with Cronbach’s α ranging from 0.925 to 0.939 and McDonald’s ω ranging from 0.927 to 0.940.

#### 2.2.2 Work interference with family (WIF)

Work interference with family (WIF) was assessed using the work-to-family conflict subscale of the Work–Family Conflict Scale developed by Netemeyer et al. (1996). The original instrument comprises two directional dimensions, namely work-to-family conflict and family-to-work conflict. The present study administered only the work-to-family conflict subscale to evaluate the extent to which officers’ job demands, working hours, and occupational strain interfered with their family lives and responsibilities. The subscale contains five items, including “The demands of my work interfere with my home and family life.” All items are rated on a seven-point Likert-type scale ranging from 1 (strongly disagree) to 7 (strongly agree). Higher scores indicate greater interference of work with family life. The scale has been translated into Chinese and has demonstrated satisfactory psychometric performance. In a previous study involving police officers, the Chinese version yielded a Cronbach’s α of 0.88 (Li et al., 2014). In the present study, the subscale exhibited high internal consistency, with both Cronbach’s α and McDonald’s ω equal to 0.934.

#### 2.2.3 Affective work rumination (AWR)

Affective work rumination (AWR) was measured using Zhang’s (2021) Chinese adaptation of the five-item affective rumination subscale from the Work-Related Rumination Questionnaire, originally developed by Cropley et al. (2012). The items capture persistent thoughts about work outside working hours that are accompanied by negative emotional arousal. One representative item is “During my nonwork time, I become tense when thinking about work-related issues.” Officers indicated how frequently they experienced each situation on a five-point Likert-type scale ranging from 1 (never) to 5 (always). Higher scores denote more frequent AWR. An earlier study involving police officers reported good internal consistency for this subscale, with a Cronbach’s α of 0.909 (Pei and Wu, 2022). In the current sample, internal consistency was similarly high (Cronbach’s α = 0.927; McDonald’s ω = 0.928).

#### 2.2.4 Copenhagen Burnout Inventory (CBI)

Occupational burnout was assessed using the Chinese version of the Copenhagen Burnout Inventory (CBI) (Kristensen et al., 2005), translated by Wu et al. (2020). The CBI is a 19-item self-report measure comprising three dimensions: personal burnout (CBI1), work-related burnout (CBI2), and client-related burnout (CBI3). These dimensions assess general physical and psychological exhaustion, exhaustion attributed to work, and exhaustion arising from interactions with service recipients, respectively. CBI1, CBI2, and CBI3 contain six, seven, and six items, respectively. Items are rated on a five-point Likert-type scale, with response options ranging from 1 (“never” or “to a very low degree”) to 5 (“always” or “to a very high degree”), depending on item wording. Higher scores indicate greater occupational burnout. Previous research reported satisfactory reliability and factor-analytic results in a Chinese nursing sample (Gao et al., 2025). In the present study, the full scale demonstrated high internal consistency, with Cronbach’s α = 0.955 and McDonald’s ω = 0.955. The corresponding coefficients were α = 0.929 and ω = 0.929 for CBI1, α = 0.933 and ω = 0.933 for CBI2, and α = 0.935 and ω = 0.936 for CBI3.

### 2.3 Statistical analysis

Data were analyzed using IBM SPSS Statistics 30.0 and IBM SPSS Amos 26.0. Descriptive statistics were first calculated in SPSS, followed by correlation and multicollinearity analyses among the study variables. Structural equation modeling (SEM) was then conducted in Amos to examine the serial mediating roles of WIF and AWR in the association between police work stress and occupational burnout.

### 2.4 Common method variance

Potential common method variance (CMV) was examined using several complementary procedures. Harman’s single-factor test was first performed on all original PCSS, WIF, AWR, and CBI items. The unrotated exploratory factor analysis identified 11 factors with eigenvalues greater than 1, collectively explaining 70.48% of the total variance. The first factor accounted for 40.00%, which was below the 50% reference threshold (Fuller et al., 2016; Howard et al., 2024; Podsakoff et al., 2003). Because Harman’s test provides only a preliminary diagnostic, additional CFA-based and collinearity assessments were conducted.

The single-factor CFA model showed poor fit, χ^2^/df = 40.002, CFI = 0.705, TLI = 0.668, RMSEA = 0.163, and SRMR = 0.108. In comparison, the hypothesized four-construct model fit the data well, χ^2^ = 376.348, df = 146, χ^2^/df = 2.578, CFI = 0.989, TLI = 0.987, RMSEA = 0.033, and SRMR = 0.022. Adding a common latent method factor produced only minor changes in model fit, χ^2^ = 355.131, df = 145, χ^2^/df = 2.449, CFI = 0.990, TLI = 0.988, RMSEA = 0.031, and SRMR = 0.025. Specifically, CFI and TLI each increased by 0.001, RMSEA decreased by 0.002, and SRMR increased by 0.003. Given the sensitivity of the chi-square statistic to the large sample size, these small changes in the alternative fit indices did not indicate a substantively meaningful improvement.

As a supplementary diagnostic, full-collinearity variance inflation factor (VIF) values were calculated by treating each focal construct in turn as an endogenous variable predicted by the remaining constructs. The resulting values ranged from 1.650 to 2.844, below the recommended threshold of 3.3 (Kock, 2015). Although CMV cannot be excluded completely because all variables were measured concurrently through self-reports, the results provided no evidence that a single method factor substantially accounted for the observed associations.

## 3 Results

### 3.1 Descriptive statistics

Descriptive statistics were first calculated for the focal variables, as presented in Table 2. The mean score for police work stress was 1.296 (SD = 0.856), with a skewness of 0.978 and a kurtosis of 2.032. Among the six PCSS dimensions, work tasks (PCSS1) had the highest mean score (M = 1.713), suggesting that task-related demands were comparatively prominent among the occupational stressors reported by the officers. Further examination of the distributions showed that the absolute skewness values for all variables were below 2 and the absolute kurtosis values were below 7, indicating that none met the conventional criteria for severe univariate nonnormality (Curran et al., 1996). The focal variables therefore showed no extreme skewness or kurtosis and were considered suitable for subsequent correlation and structural equation modeling analyses. All skewness values were positive, indicating that scores tended to cluster toward the lower end of their respective distributions.

**Table 2:** Descriptive statistics.

Variable	Mean	SD	Skewness	Kurtosis
PCSS	1.296	0.856	0.978	2.032
PCSS1	1.713	1.187	0.595	−0.072
PCSS2	1.281	1.007	0.978	1.045
PCSS3	1.110	1.082	1.168	1.227
PCSS4	1.104	1.035	1.005	0.726
PCSS5	1.159	1.142	1.037	0.740
PCSS6	1.292	1.073	0.817	0.320
WIF	3.367	1.716	0.229	−1.032
AWR	2.090	0.926	0.844	0.375
CBI	2.162	0.806	0.625	0.540
CBI1	2.272	0.939	0.465	−0.297
CBI2	2.158	0.907	0.622	−0.008
CBI3	2.056	0.943	0.749	−0.070

Because the CBI is commonly interpreted using a 0–100 scoring metric, mean CBI scores were converted to this scale and classified according to established reference thresholds (Creedy et al., 2017; Kristensen et al., 2005). As shown in Table 3, 1,233 participants (84.2%) fell below the moderate burnout threshold, 183 (12.5%) were classified as experiencing moderate burnout, 41 (2.8%) as experiencing high burnout, and 7 (0.5%) as experiencing severe burnout. Overall, 231 officers (15.8%) scored within the moderate-or-higher burnout range. These findings indicate that, although the sample as a whole reported relatively low average levels of occupational burnout, a meaningful minority of officers exhibited burnout levels warranting closer attention.

**Table 3:** Distribution of occupational burnout levels based on CBI scores.

CBI burnout category	CBI score	Frequency	Percentage (%)
Low burnout or below the threshold for moderate burnout	<50	1,233	84.2
Moderate burnout	50–74	183	12.5
High burnout	75–99	41	2.8
Severe burnout	100	7	0.5

### 3.2 Reliability, construct validity, and correlations

Composite reliability (CR) estimates, convergent and discriminant validity assessments, and correlations among the focal variables are reported in Table 4. Composite reliability values above 0.700 and average variance extracted (AVE) values above 0.500 are generally considered indicative of acceptable composite reliability and convergent validity, respectively (Fornell and Larcker, 1981). In the present study, CR values ranged from 0.927 to 0.940, all exceeding the recommended threshold of 0.700, whereas AVE values ranged from 0.566 to 0.740, all above 0.500. These results indicate satisfactory CR and convergent validity across the study constructs.

**Table 4:** Composite reliability, average variance extracted, and Pearson correlation matrix (*N* = 1,464).

Variable	CR	AVE	1	2	3	4	5	6	7	8	9	10	11
1. PCSS1	0.937	0.624	**0.790**										
2. PCSS2	0.940	0.566	0.590***	**0.752**									
3. PCSS3	0.932	0.633	0.502***	0.545***	**0.795**								
4. PCSS4	0.927	0.646	0.462***	0.528***	0.538***	**0.804**							
5. PCSS5	0.930	0.726	0.515***	0.562***	0.530***	0.570***	**0.852**						
6. PCSS6	0.932	0.603	0.552***	0.591***	0.599***	0.536***	0.536***	**0.777**					
7. WIF	0.934	0.740	0.487***	0.507***	0.437***	0.414***	0.433***	0.480***	**0.860**				
8. AWR	0.928	0.719	0.495***	0.515***	0.489***	0.503***	0.497***	0.525***	0.462***	**0.848**			
9. CBI1	0.929	0.686	0.511***	0.510***	0.499***	0.514***	0.511***	0.540***	0.517***	0.571***	**0.828**		
10. CBI2	0.933	0.666	0.524***	0.584***	0.512***	0.543***	0.527***	0.561***	0.540***	0.559***	0.665***	**0.816**	
11. CBI3	0.936	0.707	0.492^***^	0.516^***^	0.528^***^	0.520^***^	0.505^***^	0.544^***^	0.464^***^	0.505^***^	0.581^***^	0.636^***^	**0.841**

The diagonal values represent the square root of AVE, whereas values below the diagonal represent the Pearson correlation coefficients between dimensions. ***p < 0.001. CR, Composite reliability; AVE, Average variance extracted.

Discriminant validity was further assessed using the heterotrait–monotrait ratio of correlations (HTMT), with the results presented in Table 5. HTMT is considered a relatively sensitive criterion for evaluating discriminant validity. A value of 0.850 is commonly adopted as a more stringent threshold, whereas 0.900 represents a more lenient criterion (Henseler et al., 2015). In the present study, all HTMT values were below 0.850, providing further support for the discriminant validity of the measurement model.

**Table 5:** Heterotrait–monotrait ratios of correlations.

Variable	1	2	3	4	5	6	7	8	9	10
1. PCSS1	—									
2. PCSS2	0.623	—								
3. PCSS3	0.535	0.582	—							
4. PCSS4	0.491	0.564	0.579	—						
5. PCSS5	0.549	0.600	0.568	0.614	—					
6. PCSS6	0.587	0.632	0.644	0.579	0.578	—				
7. WIF	0.521	0.537	0.466	0.441	0.463	0.513	—			
8. AWR	0.531	0.549	0.524	0.539	0.532	0.565	0.497	—		
9. CBI1	0.547	0.542	0.534	0.552	0.547	0.581	0.556	0.615	—	
10. CBI2	0.561	0.620	0.548	0.581	0.564	0.601	0.578	0.601	0.714	—
11. CBI3	0.524	0.549	0.564	0.557	0.541	0.582	0.496	0.542	0.623	0.681

### 3.3 Model fit for structural equation modeling

Amos 26.0 was used to evaluate the fit of the hypothesized models. As shown in Table 6, the outcome variables were overall occupational burnout (CBI), personal burnout (CBI1), work-related burnout (CBI2), and client-related burnout (CBI3), respectively. All fit indices for the four models met commonly used criteria (Hu and Bentler, 1999; Kline, 2011), indicating that the overall burnout model and the three dimension-specific models fit the sample data well.

**Table 6:** Fit indices for the overall occupational burnout model and three dimension-specific models.

Fit index	Criterion	CBI	CBI1	CBI2	CBI3
χ^2^/df	<3	2.578	2.687	2.572	2.471
RMR	<0.08	0.037	0.039	0.038	0.039
SRMR	<0.08	0.021	0.021	0.020	0.020
GFI	>0.9	0.972	0.975	0.976	0.977
AGFI	>0.9	0.964	0.966	0.968	0.969
NFI	>0.9	0.981	0.983	0.984	0.984
RFI	>0.9	0.978	0.980	0.981	0.981
IFI	>0.9	0.989	0.989	0.990	0.991
TLI	>0.9	0.987	0.987	0.988	0.989
CFI	>0.9	0.989	0.989	0.990	0.991
RMSEA	<0.08	0.033	0.034	0.033	0.032

CBI denotes the model in which occupational burnout is specified as a second-order latent outcome. CBI1, CBI2, and CBI3 denote models in which personal burnout, work-related burnout, and client-related burnout, respectively, are specified as observed outcome scores for comparison across models. χ^2^ = chi-square statistic; df, degrees of freedom; RMR, root mean square residual; SRMR, standardized root mean square residual; GFI, goodness-of-fit index; AGFI, adjusted goodness-of-fit index; NFI, normed fit index; RFI, relative fit index; IFI, incremental fit index; TLI, Tucker-Lewis index; CFI, comparative fit index; RMSEA, root mean square error of approximation.

### 3.4 Analysis of direct effects in the structural equation models

**Figure 2:**
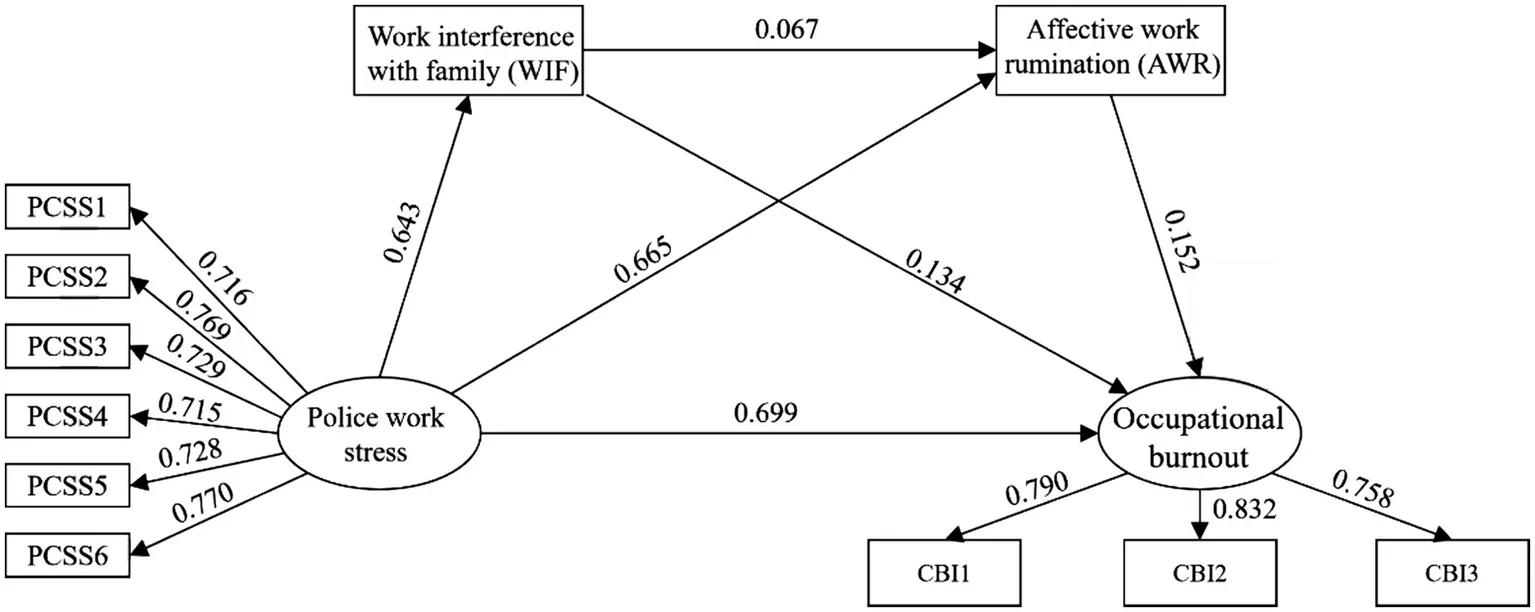
Standardized path coefficients for the hypothesized structural model. The numbers attached to the single-headed arrows reflect standardized path coefficients. All paths were significant at *p* < 0.05; All paths except WIF → AWR were significant at *p* < 0.001.

Direct path estimates for the hypothesized model are presented in Table 7 and Figure 2. Police work stress (PCSS) was significantly and positively associated with WIF (β = 0.643, *p* < 0.001), indicating that greater police work stress co-occurred with greater interference of work with family life. Police work stress was also significantly and positively associated with AWR (β = 0.665, *p* < 0.001). After police work stress was controlled, WIF remained significantly and positively associated with AWR (β = 0.067, *p* = 0.027), although this incremental association was small. Thus, WIF accounted for relatively limited incremental variance in AWR beyond police work stress. With respect to occupational burnout, police work stress was significantly and positively associated with CBI (β = 0.699, *p* < 0.001), as were WIF (β = 0.134, *p* < 0.001) and AWR (β = 0.152, *p* < 0.001).

**Table 7:** Direct path estimates for the structural equation model.

Path	B	SE	C.R.	*p*	β
PCSS → WIF	1.162	0.055	21.079	<0.001	0.643
PCSS → AWR	0.677	0.038	17.965	<0.001	0.665
WIF → AWR	0.038	0.017	2.214	0.027	0.067
PCSS→CBI	0.610	0.035	17.418	<0.001	0.699
WIF → CBI	0.065	0.012	5.308	<0.001	0.134
AWR → CBI	0.130	0.024	5.331	<0.001	0.152

B, unstandardized path coefficient; SE, standard error; C.R., critical ratio; β, standardized path coefficient.

To examine structural relations across the dimensions of occupational burnout, three supplementary dimension-specific models were estimated. The measurement and structural specifications among PCSS, WIF, and AWR were held constant, while personal burnout (CBI1), work-related burnout (CBI2), and client-related burnout (CBI3) were specified separately as outcomes. The results are presented in Table 8. The PCSS → WIF, PCSS → AWR, and WIF → AWR paths were nearly identical across the four models, indicating that the first part of the structural model was stable. PCSS was significantly and positively associated with overall occupational burnout and all three specific dimensions. WIF and AWR were significantly and positively associated with personal and work-related burnout, but neither path to client-related burnout reached statistical significance. These findings support the direct association of police work stress with each burnout outcome, while indicating dimension-specific patterns in the associations of WIF and AWR with burnout.

**Table 8:** Comparison of structural paths across the overall occupational burnout model and three dimension-specific models.

Structural path	CBI outcome			
	CBI	CBI1	CBI2	CBI3
PCSS → WIF	0.643***	0.643***	0.643***	0.643***
PCSS → AWR	0.665***	0.665***	0.664***	0.664***
WIF → AWR	0.067*	0.067*	0.067*	0.067*
PCSS→CBI outcome	0.699***	0.476***	0.572***	0.636***
WIF → CBI outcome	0.134***	0.130***	0.131***	0.042, *p* = 0.136
AWR → CBI outcome	0.152***	0.192***	0.111***	0.053, *p* = 0.093

All path coefficients are standardized estimates. Under CBI outcome, CBI denotes the hypothesized model; CBI1, CBI2, and CBI3 denote models with personal burnout, work-related burnout, and client-related burnout as the outcome, respectively. ***p < 0.001; *p < 0.05.

### 3.5 Analysis of indirect effects in the structural equation models

Bootstrapping was used to test the indirect effects. Statistical significance was determined by whether the 95% bootstrap confidence interval excluded zero (Preacher and Hayes, 2008). The results are presented in Table 9. The indirect effect of police work stress (PCSS) on occupational burnout (CBI) through WIF was significant (β = 0.086, *p* < 0.001), as was the indirect effect through AWR (β = 0.101, *p* < 0.001). In addition, the serial indirect effect through WIF and subsequent AWR reached statistical significance (β = 0.007, *p* = 0.024). The total indirect effect was significant (β = 0.194, *p* < 0.001). After WIF and AWR were included in the model, the direct effect of police work stress on occupational burnout remained significant (β = 0.699, *p* < 0.001), indicating partial mediation by WIF and AWR in the association between police work stress and occupational burnout.

**Table 9:** Bootstrap estimates of indirect effects (5,000 resamples).

Effect path	B	Boot SE	95% Bootstrap CI	*p*	β
PCSS → WIF → CBI	0.075	0.016	[0.044,0.106]	<0.001	0.086
PCSS → AWR → CBI	0.088	0.021	[0.047,0.129]	<0.001	0.101
PCSS → WIF → AWR → CBI	0.006	0.003	[0.001,0.014]	0.024	0.007
Total indirect effect	0.169	0.028	[0.113,0.224]	<0.001	0.194
Direct effect	0.610	0.036	[0.543,0.683]	<0.001	0.699
Total effect	0.780	0.023	[0.738,0.826]	<0.001	0.893

Effects were estimated using maximum likelihood and 5,000 bootstrap resamples. The 95% CI is the bias-corrected 95% bootstrap confidence interval; B = unstandardized effect; β = standardized effect.

To assess whether the indirect effects generalized across the dimensions of occupational burnout, personal burnout (CBI1), work-related burnout (CBI2), and client-related burnout (CBI3) were examined separately as outcomes and compared with the overall occupational burnout (CBI) model. The results are presented in Table 10. The direct effect of PCSS was significant for all four burnout outcomes. The total indirect effect was significant in the overall burnout, personal burnout, and work-related burnout models, but not in the client-related burnout model. Thus, the mediating roles of WIF and AWR were primarily evident for personal and work-related burnout, indicating a degree of dimension specificity.

**Table 10:** Comparison of indirect effects across overall occupational burnout and its dimensions.

Effect path	Outcome			
	CBI	CBI1	CBI2	CBI3
Total indirect effect	0.169 [0.113, 0.224]	0.242 [0.173, 0.316]	0.174 [0.104, 0.244]	0.072 [−0.005, 0.145]
Direct effect	0.610 [0.543, 0.683]	0.524 [0.437, 0.610]	0.611 [0.523, 0.700]	0.707 [0.613, 0.805]
Total effect	0.780 [0.738, 0.826]	0.766 [0.723, 0.812]	0.785 [0.743, 0.830]	0.779 [0.731, 0.830]
Mediation assessment	Partial mediation	Partial mediation	Partial mediation	Total indirect effect
	Significant	Significant	Significant	Not significant

The table reports unstandardized effects. BC 95% CI = bias-corrected 95% bootstrap confidence interval based on 5,000 resamples. Under CBI outcome, CBI denotes the hypothesized model; CBI1, CBI2, and CBI3 denote models with personal burnout, work-related burnout, and client-related burnout as the outcome, respectively.

### 3.6 Robustness analyses of the structural equation model

Some PCSS dimension definitions may overlap conceptually or in item content with WIF, AWR, and CBI. To clarify the influence of potential item-content overlap, the operational definition of PCSS was narrowed. Only PCSS1, PCSS3, and PCSS6 were retained as indicator dimensions, representing a narrower definition of PCSS. The restricted-definition model was analyzed using the same procedure as the hypothesized model to test the robustness of the findings. The model fit the data well (χ^2^/df = 2.463, CFI = 0.992, TLI = 0.990, RMSEA = 0.032, SRMR = 0.020). Table 11 shows that all direct and indirect paths remained significant, supporting the robustness of the main findings.

**Table 11:** Structural paths and effect decomposition for the narrowly defined PCSS model.

Path/Effect	B	β	P
Direct paths			
PCSS → WIF	1.167	0.645	***
PCSS → AWR	0.663	0.651	***
WIF → AWR	0.042	0.074	*
PCSS → CBI	0.585	0.665	***
WIF → CBI	0.069	0.141	***
AWR → CBI	0.154	0.179	***
Indirect paths			
PCSS → WIF → CBI	0.080	0.091	***
PCSS → AWR → CBI	0.102	0.116	***
PCSS → WIF → AWR → CBI	0.008	0.009	*
Total indirect effect	0.190	0.216	***
Total effect	0.774	0.881	***

B, unstandardized effect; β, standardized effect. Indirect effects were tested using 5,000 bias-corrected bootstrap resamples. *p < 0.05; ***p < 0.001.

In addition, to examine whether the main findings were affected by demographic differences, sex, age, years of police service, marital status, and educational level, which were collected in the original survey, were added to the hypothesized model as covariates and specified simultaneously as predictors of WIF, AWR, and CBI. Because age and years of police service were highly correlated (r = 0.969), age was standardized, whereas years of police service was represented by the standardized residual from its regression on age. The covariate-adjusted model fit the data well (χ^2^/df = 2.002, CFI = 0.988, TLI = 0.984, RMSEA = 0.026, SRMR = 0.018). As shown in Table 12, all direct and indirect paths remained significant after the covariates were included.

**Table 12:** Structural paths and effect decomposition for the demographic covariate-adjusted model.

Path/effect	B	β	P
Direct paths			
PCSS → WIF	1.190	0.659	***
PCSS → AWR	0.685	0.673	***
WIF → AWR	0.034	0.061	*
PCSS→CBI	0.625	0.716	***
WIF → CBI	0.059	0.123	***
AWR → CBI	0.128	0.149	***
Indirect paths			
PCSS → WIF → CBI	0.071	0.081	***
PCSS → AWR → CBI	0.088	0.101	***
PCSS → WIF → AWR → CBI	0.005	0.006	*
Total indirect effect	0.164	0.188	***
Total effect	0.789	0.904	***

B, unstandardized effect; β, standardized effect. Indirect effects were tested using 5,000 bias-corrected bootstrap resamples. *p < 0.05; ***p < 0.001.

As shown in Table 13, among the covariate paths, age was associated with lower WIF (β = −0.069), whereas female sex was associated with higher WIF (β = 0.085, *p* < 0.001) and higher CBI (β = 0.063, *p* < 0.001). None of the remaining covariate paths reached statistical significance. These results indicate that the main structural associations remained relatively stable after adjustment for the demographic covariates measured in the study.

**Table 13:** Standardized path estimates from demographic covariates to WIF, AWR, and CBI.

Covariate and coding/comparison	WIF β(p)	AWR β(p)	CBI β(p)
Age (Standardized)	−0.069*	−0.035(0.135)	0.004(0.828)
Years of police service (age residual, standardized)	−0.033(0.137)	−0.005(0.803)	−0.013(0.455)
Female (male = reference)	0.085***	0.018(0.395)	0.063***
Married (not married = reference)	0.020(0.420)	−0.012(0.599)	−0.014(0.483)
High school or below (bachelor’s degree = reference)	0.000(0.996)	0.008(0.719)	−0.021(0.217)
Junior college (bachelor’s degree = reference)	−0.035(0.120)	0.019(0.372)	−0.011(0.548)
Master’s/doctoral degree (bachelor’s degree = reference)	−0.011(0.608)	0.018(0.387)	−0.004(0.827)

*p < 0.05; ***p < 0.001.

## 4 Discussion

### 4.1 Police work stress and occupational burnout

The study found a significant positive association between police work stress and occupational burnout, supporting H1. After WIF and AWR were included simultaneously, police work stress remained significantly and positively associated with overall occupational burnout and with personal, work-related, and client-related burnout. This pattern is consistent with previous findings (Alves et al., 2023; Purba and Demou, 2019; Zhou et al., 2024). The present study further shows that the association persists when exhaustion is differentiated by its contextual referent. Greater police work stress co-occurred with general physical and psychological exhaustion and with exhaustion attributed to work as a whole, indicating a broad cross-sectional association between policing stress and experiences of exhaustion. These findings suggest that the relation between police work stress and occupational burnout may usefully be understood as a process of risk accumulation and as a potential early-warning signal. In burnout prevention, work stress can serve as an upstream indicator for screening and organizational intervention. Police organizations could develop stress-management pathways around task allocation, recovery between shifts, support for operational risks, organizational communication, and psychological services to reduce the likelihood that persistent high-stress states develop into occupational burnout.

### 4.2 The independent mediating role of work interference with family

The indirect association between PCSS and CBI through WIF was statistically significant, supporting H2. Prior research has closely linked work–family conflict with psychological strain, emotional exhaustion, and occupational burnout (Amstad et al., 2011; Griffin and Sun, 2018). When working hours and work stress interfere with family responsibilities, family activities, and family relationships, individuals may experience fewer opportunities for rest, restricted family interaction, and accumulating role strain (Greenhaus and Beutell, 1985; Netemeyer et al., 1996; Thompson et al., 2005). These life-role experiences remained associated with occupational burnout after AWR was included, suggesting that officers’ off-job exhaustion cannot be fully reduced to continued thinking about work.

The dimension-specific models showed that WIF was significantly and positively associated with personal and work-related burnout, but not with client-related burnout. Longitudinal research among police officers suggests that work–family conflict and emotional exhaustion may form an interrelated process of change (Hall et al., 2010). Work–family conflict is consistently related to emotional exhaustion, job dissatisfaction, and physical and psychological strain, and WIF may have stronger matching-domain associations with work-related outcomes (Allen et al., 2000; Amstad et al., 2011; Kossek and Ozeki, 1998). When officers return home to delayed family responsibilities, altered family activities, and reduced intimate communication caused by work, the restorative function of family life may decline, making it more difficult to replenish depleted resources through rest, companionship, and emotional support (Moreno-Jiménez et al., 2009; Sonnentag et al., 2010; Thompson et al., 2005). At the same time, disrupted family responsibilities and reduced opportunities for recovery correspond more directly to general physical and psychological exhaustion and exhaustion attributed to work as a whole, whereas client-related burnout may depend more strongly on the emotional demands and conflictual encounters involved in law-enforcement interactions (Kristensen et al., 2005; McCreary and Thompson, 2006), resulting in a distinct pattern of associations.

The findings also show that WIF accounted for a limited proportion of the association in the hypothesized model: its specific indirect effect was smaller than the direct association retained between police work stress and occupational burnout. Prior research indicates that heavy workloads, role conflict, organizational pressure, exposure to danger, and persistent emotional demands can themselves deplete psychological and physiological resources. Even before these stressors visibly spill over into family life, they may be directly associated with emotional exhaustion and the gradual development of burnout (Galanis et al., 2021; Maria et al., 2018). WIF may therefore capture only the specific resource-loss process that occurs when work stress crosses the work-family boundary, rather than all pathways linking police work stress with occupational burnout.

These findings suggest that WIF can serve as an indicator of stress spillover and impaired recovery in efforts to prevent police burnout. Police organizations could reduce the risk that work stress is associated with burnout by improving shift and recovery arrangements, limiting unnecessary overtime, strengthening family support, and enhancing work-home boundary management.

### 4.3 The independent mediating role of affective work rumination

The specific indirect association between PCSS and CBI through AWR was statistically significant, supporting H3. Prior research shows that greater job demands are commonly accompanied by more AWR and that negative work-related thoughts during nonwork time are closely associated with burnout, emotional exhaustion, work-related fatigue, and poorer sleep quality (Jimenez et al., 2022; Pei and Wu, 2022; Perko et al., 2017; Querstret and Cropley, 2012).

The dimension-specific models showed that AWR was significantly and positively associated with personal and work-related burnout, but not with client-related burnout. Repeatedly thinking about a stressful event may prolong emotional and physiological activation after the event has ended, and AWR has been linked with poorer sleep, work-related fatigue, and emotional exhaustion (Brosschot et al., 2006; Jimenez et al., 2022; Querstret and Cropley, 2012). The nonsignificant association between AWR and client-related burnout may reflect a difference in construct referents: AWR encompasses negative thinking about work problems in general, whereas client-related burnout requires officers to attribute exhaustion specifically to their work with service recipients. Client-related burnout may be more directly associated with emotional demands specific to service interactions, particularly requirements to conceal emotions (Kristensen et al., 2005). Thus, persistent negative work-related thinking during nonwork time appears to be related primarily to general fatigue and exhaustion attributed to work as a whole, but may be insufficient to explain exhaustion specifically attributed to interactions with service recipients. The latter may be more closely associated with proximal interpersonal demands such as interaction frequency, conflict intensity, and emotional labor (Pei and Wu, 2022; Queirós et al., 2020).

Analysis of the hypothesized model showed a relatively strong association between police work stress and AWR, but the incremental association between AWR and occupational burnout was small; the specific indirect effect through AWR was also smaller than the direct association retained between police work stress and burnout. Prior research indicates that work stress may continue into nonwork time as sustained work-related cognitive activity, with different forms of work stress showing stable but time-dependent associations with AWR (Zhang et al., 2024), while AWR is consistently linked with fatigue, impaired sleep, and poorer mental health (Querstret and Cropley, 2012). However, this association does not imply that AWR independently accounts for most of the process linking work stress with burnout. One possible reason is that the AWR measure captures not only whether individuals continue to think about work, but also salient negative emotional and fatigue experiences, such as feeling tense, irritated, distressed, or tired when thinking about work (Cropley et al., 2016; Pauli et al., 2023). These experiences are phenomenologically proximate to occupational burnout, particularly the fatigue and resource-depletion components of emotional exhaustion. Longitudinal research also suggests that the relation between work rumination and exhaustion is not simply unidirectional. Perko et al. (2017) found substantial correspondence between work-related rumination and exhaustion at both between-person and within-person levels: groups with high rumination generally also reported high exhaustion, while decreases in rumination co-occurred with improvements in exhaustion. Research on work recovery further indicates that difficulty psychologically detaching from work is consistently related to fatigue and exhaustion, but this relation may involve a continuing resource-loss cycle and cannot be explained solely in terms of rumination leading to exhaustion (Sonnentag and Fritz, 2015; Wendsche and Lohmann-Haislah, 2017). Existing fatigue and exhaustion may also impair cognitive control and recovery capacity, making it more difficult to stop repetitive work-related thinking during nonwork time (Weiher et al., 2022).

These findings suggest that AWR may serve as an indicator of impaired psychological recovery in efforts to prevent police burnout. Police organizations could reduce the likelihood that officers remain caught in persistent negative work-related thinking during nonwork time by providing psychological detachment training, post-work recovery support, emotion-regulation training, and structured support for processing stressful incidents.

### 4.4 The serial mediating roles of work interference with family and affective work rumination

The prespecified PCSS → WIF → AWR → CBI serial indirect association was statistically significant, supporting H4. When work stress enters the family domain, it may constrain resources available for family roles, impair recovery conditions, and increase continued cognitive processing of work events (Ten Brummelhuis and Bakker, 2012; Demerouti et al., 2004; Ilies et al., 2007). WIF also positively predicted AWR. WIF occurs when job demands, working hours, and work stress intrude on family responsibilities and activities (Greenhaus and Beutell, 1985; Netemeyer et al., 1996). AWR may then translate the incomplete recovery associated with WIF into higher occupational burnout. Persistent rumination can prolong the psychological and physiological activation associated with work stress, impede resource replenishment during nonwork time, and allow fatigue and negative affect to accumulate (Brosschot et al., 2006; Kristensen et al., 2005; Querstret and Cropley, 2012).

The serial indirect effect through WIF and AWR was small. Thus, although police work stress may intrude into the family domain through job demands, persist as negative work-related thinking during nonwork time, and ultimately be associated with greater burnout risk, this cross-domain and cross-temporal resource-loss process accounts for only part of the association between work stress and burnout. Prior research has found a stable association between work-nonwork conflict and exhaustion (Reichl et al., 2014). Among correctional officers, whose work shares the highly demanding features of policing, work-life conflict and greater AWR have likewise been associated with more severe emotional exhaustion (Kinman et al., 2017). Negative work-related rumination also prolongs the psychological representation of work stress during nonwork time, interferes with psychological detachment and resource recovery, and is consistently related to fatigue and exhaustion (Jimenez et al., 2022; Perko et al., 2017; Querstret and Cropley, 2012). However, a serial indirect effect involves several successive links, each transmitting only part of the stress association; its overall magnitude is therefore typically smaller than more proximal direct or single indirect effects. This pattern also suggests that WIF and AWR are not the only mechanisms through which police work stress is associated with burnout.

Dimension-specific analyses further showed that the total indirect effect was significant for personal and work-related burnout but not for client-related burnout. This difference may reflect variation in the sources of the three burnout dimensions. WIF and AWR primarily capture continuing resource depletion after work stress crosses the work boundary. They may therefore accumulate more readily as general fatigue and exhaustion attributed to work, producing more stable associations with personal and work-related burnout. In contrast, client-related burnout has a more specific relational referent and may depend more strongly on proximal interpersonal stressors arising from direct interactions with service recipients during law enforcement, including emotional dissonance, conflict, aggression, and mistreatment. Research likewise indicates that emotional dissonance and mistreatment by service recipients are more directly related to exhaustion within service relationships (Baranik et al., 2017; Nielsen et al., 2023).

These findings suggest that interventions addressing police burnout should attend simultaneously to the extension of work stress into the family domain and nonwork time, reduce the encroachment of work on family responsibilities and recovery opportunities, and help officers reduce the continued activation of negative work-related thoughts.

## 5 Implications and limitations

### 5.1 Implications

This study examined the association between police work stress and occupational burnout and the mediating roles of WIF and AWR. It extends research on burnout risk processes among police officers and has implications for reducing burnout risk. Theoretically, police work stress was positioned as an upstream stress source, occupational burnout as an outcome associated with continuing resource depletion, and WIF and AWR as two mediating mechanisms linking them. WIF captures the cross-domain process through which work stress extends into family life, whereas AWR captures the psychological process through which work stress maintains cognitive and emotional activation during nonwork time. Placing both variables within a single model makes it possible not only to distinguish two specific indirect associations, but also to test the joint serial indirect association they form. The study thereby refines the resource-depletion account of the association between police work stress and occupational burnout and extends this theoretical interpretation to the occupational context of Chinese policing.

Practically, the findings first identify police work stress as an important predictor of occupational burnout. Burnout-prevention programs in police organizations should incorporate work stress monitoring into routine occupational health management, with particular attention to task demands, shift scheduling and recovery, organizational communication, support for operational risks, and psychological services. In practice, more balanced personnel allocation, reductions in unnecessary administrative burdens, and improved psychological support and recovery arrangements following high-risk duties may help limit the depletion of officers’ physical and psychological resources under sustained high stress. The findings also identify WIF and AWR as important process factors associated with burnout. Police organizations could strengthen family-supportive management through flexible compensatory leave, support for family communication, and family-care arrangements following major operations, helping officers maintain essential family-role functioning. Psychological services could provide training in psychological detachment, emotion regulation, sleep recovery, and structured processing of stressful incidents. Such training may help officers reduce persistent negative work-related thinking after their shifts, respond more objectively to stressful events, and make better use of nonwork time for recovery, thereby increasing the practical value of stress management for burnout prevention.

### 5.2 Limitations

First, the conclusions are based on questionnaire data obtained primarily through self-report. Future research could integrate informant reports, organizational records, interviews, physiological indicators, and longitudinal logs to examine the association between police work stress and occupational burnout more comprehensively. Second, WIF and AWR partially mediated the association between police work stress and occupational burnout. Other intermediary variables may also be involved; future studies could examine the combined roles of psychological detachment, social support, sleep quality, emotion regulation, and organizational justice. Finally, the cross-sectional design precludes conclusions about the temporal ordering and causal direction of police work stress, WIF, AWR, and occupational burnout. Although the findings are consistent with the sequence proposed in this study, reverse or reciprocal relations may exist between WIF and AWR, and burnout may also influence perceptions of work stress and work-family interference. Future research could use multiwave longitudinal designs, diary methods, or experience sampling to compare the proposed, reverse, and reciprocal sequences and thereby identify the temporal structure of these associations more accurately.

## 6 Conclusion

Using a sample of active-duty police officers from a province in southeastern China, this study examined the association between police work stress and occupational burnout and the mediating roles of WIF and AWR. After both mediators were included, a strong direct association between police work stress and occupational burnout remained. The two independent indirect effects through WIF and AWR were each larger than their serial indirect effect. The total indirect effect through the two mediators was significant in the personal and work-related burnout models, but not in the client-related burnout model. The findings indicate that police burnout involves a continuing process of resource depletion by work stress, spillover from the work role into the family role, and persistent negative work-related thinking during nonwork time. Future police occupational health management should attend simultaneously to work stress control, family-support provisions, psychological detachment training, and the regulation of AWR to reduce the risk that work stress accumulates into occupational burnout.

## Data Availability

The raw data supporting the conclusions of this article will be made available by the authors, without undue reservation.
